# Metabolic Targets for Improvement of Allogeneic Hematopoietic Stem Cell Transplantation and Graft-vs.-Host Disease

**DOI:** 10.3389/fimmu.2019.00295

**Published:** 2019-03-05

**Authors:** Natalia M. Tijaro-Ovalle, Theodoros Karantanos, Hong-Tao Wang, Vassiliki A. Boussiotis

**Affiliations:** ^1^Division of Hematology-Oncology, Beth Israel Deaconess Medical Center, Harvard Medical School, Boston, MA, United States; ^2^Department of Medicine, Beth Israel Deaconess Medical Center, Harvard Medical School, Boston, MA, United States

**Keywords:** T cells, antigen presenting cells (APCs), GVHD, metabolism, GVL

## Abstract

Utilization of the adaptive immune system against malignancies, both by immune-based therapies to activate T cells *in vivo* to attack cancer and by T-cell therapies to transfer effector cytolytic T lymphocytes (CTL) to the cancer patient, represent major novel therapeutic advancements in oncologic therapy. Allogeneic hematopoietic stem cell (HSC) transplantation (HSCT) is a form of cell-based therapy, which replaces the HSC in the patient's bone marrow but also serves as a T-cell therapy due to the Graft-vs.-leukemia (GVL) effect mediated by donor T cells transferred with the graft. Allogeneic HSCT provides one potentially curative option to patients with relapsed or refractory leukemia but Graft-vs.-Host-Disease (GVHD) is the main cause of non-relapse mortality and limits the therapeutic benefit of allogeneic HSCT. Metabolism is a common cellular feature and has a key role in the differentiation and function of T cells during the immune response. Naïve T cells and memory T cells that mediate GVHD and GVL, respectively, utilize distinct metabolic programs to obtain their immunological and functional specification. Thus, metabolic targets that mediate immunosuppression might differentially affect the functional program of GVHD-mediating or GVL-mediating T cells. Components of the innate immune system that are indispensable for the activation of alloreactive T cells are also subjected to metabolism-dependent regulation. Metabolic alterations have also been implicated in the resistance to chemotherapy and survival of malignant cells such as leukemia and lymphoma, which are targeted by GVL-mediating T cells. Development of novel approaches to inhibit the activation of GVHD-specific naïve T cell but maintain the function of GVL-specific memory T cells will have a major impact on the therapeutic benefit of HSCT. Here, we will highlight the importance of metabolism on the function of GVHD-inducing and GVL-inducing alloreactive T cells as well as on antigen presenting cells (APC), which are required for presentation of host antigens. We will also analyze the metabolic alterations involved in the leukemogenesis which could differentiate leukemia initiating cells from normal HSC, providing potential therapeutic opportunities. Finally, we will discuss the immuno-metabolic effects of key drugs that might be repurposed for metabolic management of GVHD without compromising GVL.

## Introduction

Quiescent immune cells use glucose, amino acids, and lipids to meet their bioenergetic demands. ATP, the key energy-transporting molecule, is generated in every cell during the breakdown of such nutrients by glycolysis and OXPHOS. Depending on the functional demands, cell metabolism can be shifted toward anabolic reactions leading to production of molecules involved in biosynthesis necessary for cell growth, or toward catabolic reactions leading to breakdown of macromolecules and the generation of byproducts, which are subsequently used for energy generation or for construction of anabolic pathways. A balance of these anabolic and catabolic processes is mandatory for maintenance of metabolism homeostasis (1).

Glucose is the most abundant extracellular nutrient and, although ATP production during glucose catabolism by glycolysis is significantly lower compared to the ATP generated by OXPHOS reactions, it is faster and more efficient in increasing cellular ATP than mitochondrial metabolism. Glycolysis also supports cell growth because glycolytic intermediates provide a bridge to multiple biosynthetic pathways, including PPP that has an important role in building blocks necessary for nucleotide biosynthesis, rapid generation of metabolic intermediates, and cell growth (2, 3). Additionally, glycolysis fuels production of NADPH, which is mandatory not only for the support of anabolic pathways but also plays a crucial role in decreasing the oxidative stress in rapidly proliferating cells and maintaining the redox state of the cell (4). Pyruvate derived from glucose in glycolysis can be converted into lactate in the cytoplasm or into acetyl-CoA in the mitochondria to subsequently enter the TCA cycle (also known as Krebs cycle). In addition to producing intermediates that feed multiple biosynthetic pathways, the oxidative reactions of the TCA cycle generate NADH and FADH_2_ which are required for the donation of electrons to the electron-transport chain for OXPHOS.

Rapidly proliferating malignant cells preferentially use glucose to sustain their rapid growth in the hazardous TME (5). The preference of cells to ferment glucose to lactic acid, even in the presence of oxygen that could support OXPHOS, is known as the Warburg effect (6). Although originally observed in cancer cells, it is now known that the Warburg effect is used by most cell types, including immune cells, to generate energy during times of rapid growth, because using glucose for energy generation through glycolysis, spares other nutrients for usage in anabolic reactions.

**Figure 1:**
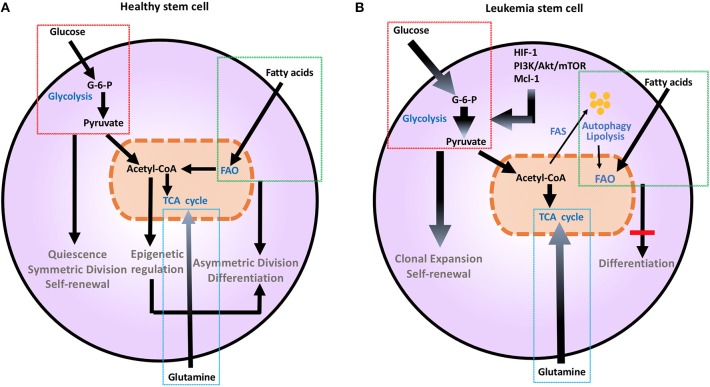
Metabolic changes in healthy **(A)** and leukemia stem cells **(B)**. Metabolic changes drive stem cell differentiation in healthy subsets and clonal expansion in leukemia stem cells. Glucose uptake and glycolysis supports pluripotency and self-renewal of HSC, and is associated to the persistent ability of HSC to engage glycolysis, converting glucose to G-6-P and pyruvate, while sustaining a low ATP state. During differentiation, HSCs engage mitochondrial metabolic programs, including TCA cycle and FAO. This shift toward more efficient ATP generation is important for maturation and long-term survival. Mutations and aberrant protein expression related to malignant conversion of HSCs, including upregulation of HIF-1, PI3K/Akt/mTOR, and Mcl-1, induce metabolic changes. Leukemia stem cells support their uncontrolled clonal expansion by significantly increasing glucose uptake and metabolism (thick arrows). Although LSCs are highly dependent on glycolysis under steady state conditions, they have a high degree of metabolic plasticity and adaptation potential and can utilize autophagy and catabolic pathways such as OXPHOS and FAO in the setting of energy stress to support their survival and proliferation. Glutamine addiction is also an important feature common to malignant hematopoietic cells (thick arrow). Together these changes support extensive self-renewal without differentiation of LSC. Akt, Protein Kinase B; ATP, Adenosine triphosphate; G-6-P, Glucose-6-phosphate; FAO, Fatty acid oxidation; HIF-1, hypoxia induced factor 1; HSCs, hematopoietic stem cells; Mcl-1, myeloid cell leukemia 1; mTOR, Mechanistic/mammalian target of rapamycin; PI3K, Phosphatidylinositol-4,5-bisphosphate 3-kinase; TCA, Tricarboxylic acid cycle.

Metabolic aberration provides a key signature that differentiates malignant hematopoietic cells from normally differentiating hematopoietic progenitors that give rise to committed progenitors and mature myeloid cells (7). As in other cancer types, the Warburg effect dominates the metabolic preference of leukemia cells (7, 8), whereas during normal HSC differentiation glycolysis declines and mitochondrial metabolism and FAO gradually increases (9) (Figure 1). It has been hypothesized that leukemia cells that are resistant to treatment and responsible for relapses, have features of “LSC” that have the ability to reproduce the disease in animal models (10). These LSC, also known as leukemia initiating cells, appear to have unique metabolic features that differentiate them not only from normal HSCs but also from other leukemia cells. These findings underline the significance of metabolism in leukemia initiation and relapse.

AlloHSCT provides the only curative therapeutic approach for aggressive leukemias and lymphomas that are resistant to chemotherapy and immunotherapy. One of the key benefits of this therapy comes directly from the T cell-mediated offense to cancer, a process known as GVL effect (11). Nonetheless, T lymphocytes can also drive GVHD, the principal cause of non-relapse mortality among alloHSCT recipients. GVHD results from the attack of healthy recipient tissue by donor T cells that recognize host's alloantigens. Detailed, extensive studies have identified that T cells involved in GVHD are substantially different from the ones that mediate GVL (12–14). Specifically, naïve αβ TCR-positive T cells appear to be the main mediators of GVHD upon activation by host antigens (15). Conversely, T_MEM_ subsets have been found to sustain GVL function (12–14), suggesting that the immunologic and metabolic mechanisms implicated in these two effects after allotransplantation are distinct. Moreover, Treg also play a role in GVHD and GVL as they have the ability to suppress GVHD without compromising GVL (16). As a consequence, Tregs have been considered a therapeutic target for the control of GVHD either as a cell-based immunotherapy (17, 18) or as an *in vivo* therapeutic target by using approaches that induce Treg differentiation and expansion (19, 20).

GVHD is the leading cause of non-relapse mortality after HSCT because its prevention and treatment remain challenging. Global immunosuppression is the mainstay of therapy for GVHD but responses are only partial in most cases. Moreover, complications of chronic immunosuppression are detrimental (21, 22). As an alternative, the administration of T cell depleted donor grafts has been tested, but the high relapse and infection rates seen in patients who receive these graft variants mostly guide against the use of this strategy (23). This renders the discovery of new strategies that can ameliorate GVHD, while preserving the benefits from GVL effect, a real necessity.

Metabolism is an attractive tentative target for therapeutic intervention both in cancer immunotherapy and GVHD. T cell subsets are poised to distinct metabolic pathways that can determine their function and differentiation (24, 25). Upon activation, naïve T cells rely on glycolytic metabolism to rapidly meet the bioenergetic needs required for their proliferation, TCR rearrangement, production of growth factors, and differentiation to T_EFF_. On the contrary, the function of Treg and T_MEM_ cells depends on enhanced FAO (26, 27). Because distinct T cell subsets mediate GVHD vs. GVL, the dominant metabolic properties of these distinct subsets might serve as new therapeutic targets that can be exploited for prevention or suppression of GVHD without compromising GVL.

Although in the context of GVHD and GVL, emphasis has been placed on T cells, the innate immune cells of the host, particularly macrophages and dendritic cells, have an indispensable role in the activation of alloreactive T cells (28–31). Differentiation, proliferation and function of innate immune cells are also subjected to metabolism-dependent regulation (3). After allogeneic HSCT, these components of the immune system function in the context of the engrafted and rapidly expanding allogeneic HSC, residual leukemia cells potentially remaining at the state of MRD and rapidly dividing cells in host non-hematopoietic tissues that are the targets of GVHD, such as the gut (32, 33).

Based on the above, it is apparent that targeting metabolism for therapy of GVHD will require thorough understanding of the unique metabolic properties and programs of the multiple cellular components involved in GVHD and GVL. In the following sections we will briefly highlight the metabolic features of malignant hematopoietic cells and we will discuss the metabolic features that guide the function of T cells and APCs during processes involved in GVHD and GVL. We will also provide rationale for potential therapeutic interventions by targeting metabolic pathways that guide the differentiation and function of these immune cells in the context of alloHSCT.

## Metabolism in Normal and Malignant Hematopoietic Cells

Metabolic changes drive division and differentiation of HSC and MP (9). HSCs are predominantly quiescent, in G_0_ phase, but divide approximately every 145 days, as a consequence of a cell-cycle-linked maturation process (34, 35). Their dormancy is important to sustain adult HSC pluripotency and to prevent HSC exhaustion (36). In order to maintain this state, HSCs utilize aerobic glycolysis and suppress oxidative phosphorylation, thereby maintaining an environment of low ROS (37). HSCs respond rapidly to stimuli to either maintain themselves via self-renewal by sustaining glycolytic metabolism and symmetric division or give rise to committed progenitors, by shifting their metabolism toward mitochondrial metabolism and activation of TCA cycle or FAO and asymmetric division (9) (Figure 1A). This is supported by the observation that depleting the mitochondrial oxidative phosphatase *PTPMT1* blocks the entry into the cell cycle and differentiation of HSC (38). Maturation from a pluripotent state to a committed progenitor state also requires precise epigenetic modifications (39). Defects in DNA methyltransferases Dnmt3a and Dnmt3b that regulate such epigenetic effects are associated with impaired stem cell differentiation, leading to leukemia-inducing events (40).

Similarly to other malignant cell types, *anabolic metabolism* is the signature of malignant hematopoietic cells including AML, MM, and ALL (7, 41–43). This is mediated by upregulation of glucose transporters and increase of glucose uptake and glycolysis. Such changes are induced by molecular aberrations and inappropriate activation of signaling pathways such as PI3K/Akt/mTOR, enhanced pro-survival mechanisms, and hypoxia (5). Normal and malignant hematopoietic cells also highly depend on the use of glutamine. This is related to the expression of *myc*, which is proportional to HSC multipotency, cell-maintenance, and self-renewal (44). Upregulation of c-*myc* in high-grade lymphomas increases glutaminolysis and leads to glutamine dependence and addiction of malignant cells to support their biosynthesis pathways. Anaplerosis via glutamine usage in the TCA cycle may be a c-*myc*-mediated mechanism critical for survival and growth of malignant hematopoietic cells (45). An additional important anabolic pathway in malignant hematopoietic cells is fatty acid synthesis. Non-Hodgkin B-cell lymphoma cells are particularly sensitive to C-75, a fatty acid synthase inhibitor, supporting the premise that rapidly proliferative lymphoma cells are not only dependent on aerobic glycolysis but on other anabolic pathways for their growth and proliferation (46).

LSCs, which are responsible for survival and persistence of leukemia, are more dependent on aerobic glycolysis (Figure 1B) and display higher expression of the glycolysis enzymes PKM2 and LDH-A compared to normal HSCs. In turn, combined inhibition of PKM2 and LDH-A leads to eradication of LSCs (7). LSCs also rely on *catabolic pathways* for the production of energy and can utilize fatty acids for FAO in order to escape the detrimental effects of chemotherapy and maintain their survival under conditions of stress (47). Deletion of AMPK, an important sensor of energetic stress that maintains metabolic homeostasis by activating catabolic metabolism and autophagy, synergizes with metabolic stress caused by nutrient restriction in LSCs and profoundly suppresses leukemogenesis (48). In CML, autophagy acts as a possible mechanism of survival and resistance of leukemia to TKI treatment (49). Under these conditions, inhibition of mitochondrial OXPHOS can eradicate TKI-resistant CML LSCs (50). Thus, although LSCs are highly dependent on glycolysis under steady state conditions, they have a high degree of metabolic plasticity and adaptation potential and can utilize catabolic pathways in the setting of energy stress to support their survival and proliferation. The clinical relevance of the increased metabolic plasticity that is pivotal in LSCs is supported by the observation that BCL-2 blockage, which reduces OXPHOS, selectively eliminates this quiescent leukemia subset (51).

Our knowledge regarding the metabolic features of leukemia cells in relapsed or resistant disease in patients who undergo allogeneic HSCT is limited because relevant studies are currently missing. As mentioned above, relapsed or resistant leukemia cells display features of LSC, which are highly depend on glycolysis but also have the metabolic plasticity to adopt other metabolic programs for energy generation, including mitochondrial metabolism, FAO and autophagy. Thus, although therapeutic approaches to target glycolytic metabolism to inhibit activation of GVHD-mediating T_EFF_ cells are expected to suppress or eradicate MRD, it is possible that plasticity and metabolic adaptation will allow LSC to survive by shifting their metabolic preferences. Focused studies are required to address this issue.

## Immuno-Metabolic Reprograming and Hematopoietic Stem Cell Transplantation

### Role of Metabolism in T Cell Differentiation and Relevance to Alloreactive T Cell Function

Resting T cells rely on mitochondrial respiratory capacity and OXPHOS for their metabolism and bioenergetic demands. Upon activation, they demand higher energetic supply, met mostly by the engagement of glycolytic pathway and mitochondrial OXPHOS (52). Similar to cancer cells, activated T cells predominantly depend on glycolysis for energy production and generation of biosynthetic intermediates while sparing other nutrients for anabolic reactions. Glycolysis has a key role in the differentiation of T effector cells. Conversely, glucose deprivation impairs the ability of CD8^+^ T cells to express IFN-γ gene, a signature of their differentiation into the effector state (53). Extracellular glucose that T cells uptake during the effector phase, supports fatty acid *de novo* synthesis and these newly synthesized lipids form the fuel used after the transition and differentiation of T_EFF_ to T_MEM_ cells (27). Environmental cues that promote T_MEM_ cell differentiation, such as IL-15, promote mitochondrial biogenesis and the expression of Cpt1a, which allows entry of long chain fatty acids to the mitochondria and functions as the rate limiting enzyme for FAO. These immune-metabolic properties are associated with longevity and survival in high-stress environments (54). In contrast, pathologically activated lymphocytes, such as those in autoimmune diseases, activate mitochondrial metabolism but utilize glucose for OXPHOS (55).

Metabolic pathways are also linked to the functional differentiation and polarization of T cell subsets. Th1, Th2, Th17 and Tfh preferentially undergo glycolysis by increasing the expression of Glut1 and by activating the PI3K/Akt/mTOR pathway (26). mTOR plays a role as a cell nutrient sensor and is a crucial regulator of T cell metabolism (56) by activating anabolic reactions including glycolysis, but also fatty acid metabolism, by targeting SREBPs (57). Through these coordinated effects, mTORC1 leads to Th1 and Th17 differentiation along with regulation of T cell priming and generation of iTregs, while mTORC2 drives differentiation to Th2 (58). Although Th17 cells are known to depend on glycolysis (59), inhibiting ACC1, a key mediator for *de novo* fatty acid synthesis, impairs Th17 development in both human and mouse models, favoring the formation of anti-inflammatory Foxp3^+^ Tregs (60). The significance of these complex effects mediated by mTOR on pathways that regulate glycolysis and fatty acid metabolism are also supported by the implications induced on T cell differentiation and function by AMPK signaling (61) which negatively regulates mTOR-mediated glycolytic metabolism (62). AMPK promotes FAO by multiple mechanisms, including the direct regulation of key lipid metabolizing enzymes, the negative regulation of the mTOR and the intracellular transport of fatty acids (63–65). These coordinated processes, leading to lipid synthesis and utilization, provide two key properties of T_MEM_ cells, namely longevity and immune quiescence (66).

It is therefore apparent that mTOR actively influences the differentiation of all T cell subsets that are involved in GVHD, including Th1, Th2, Th17 and Tfh cells. Th1, Th2 and Th17 have essential roles in the induction of aGVHD, while Tfh cells are pathogenic in cGVHD (67). Intriguingly, Tregs and T_MEM_ cells, which appear to be protective from GVHD, also depend on mTOR for their differentiation and function (68, 69). Due to their overall inhibitory effect on T effector cell function, mTOR antagonists such as sirolimus are routinely used for the prophylaxis or treatment of GVHD in alloHSCT recipients (70). The addition of RGI-2001, a synthetic CD1 ligand that expands Tregs *in vivo*, to sirolimus results in a greater decrease in GVHD rates, as compared to the ones achieved by either compound alone (71).

Although these results indicate that mTOR antagonists support the activation and differentiation of Treg *in vivo*, the mechanistic role of mTOR in Treg biology remains controversial. The absence of mTORC1 signaling during T cell differentiation has been associated with lack of Th1/Th2 polarization and enhanced conversion to Treg phenotype (58). Surprisingly, conditional targeting of mTORC1 in Treg cells by deletion of the mTORC1 partner, Raptor, resulted in impaired fatty acid and cholesterol synthesis, leading to defective Treg generation and function (72). Conversely, the absence of mTORC2 signaling by deletion of the mTORC2 partner, Rictor, potentiated the generation of short-lived effector and memory precursor CD8^+^ T cells (73). The combined administration of the mTOR inhibitor, Rapamycin, and IL-2 not only preserved but also promoted Treg expansion and increased the donor CD4^+^ CD25^+^ Foxp3^+^ Tregs, resulting in decreased aGVHD-related mortality (74). This implies that Treg differentiation and function is positively regulated by mTOR inhibition and mTOR-independent IL-2-mediated signaling. Conversely, cyclosporin, which inhibits IL-2 production by targeting NFAT signaling, compromised Treg proliferation *in vivo* (68). In light of their specific effects on Treg differentiation and expansion, the mechanisms of these immunosuppressive agents in the prevention and treatment of GVHD should be revisited and their Treg-dependent immunoregulatory effects should be considered when these compounds are used for the prevention or treatment of GVHD.

### Metabolism of Alloactivated GVHD-Mediating T Cells

**Figure 2:**
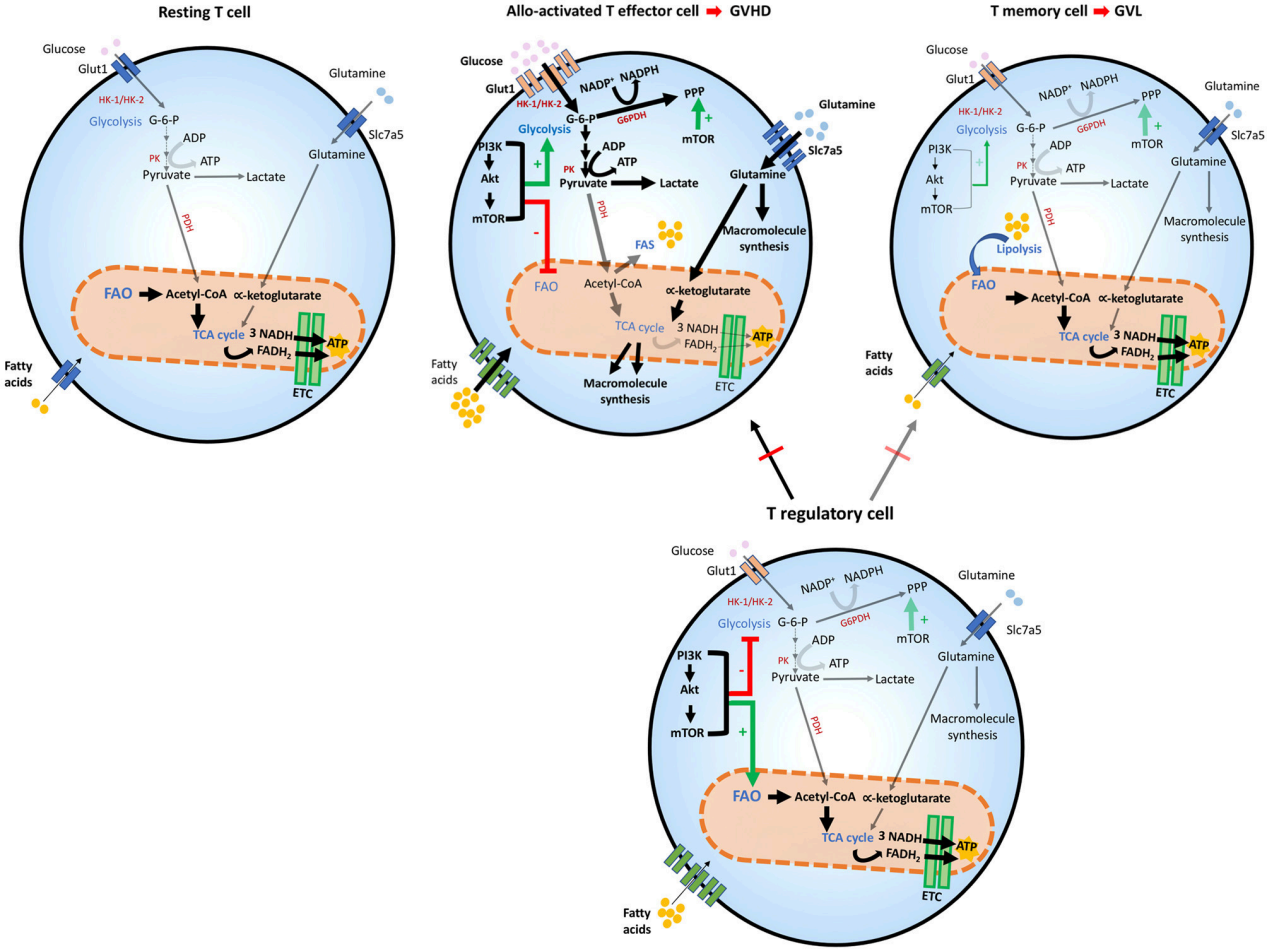
Metabolic reprogramming of T cells after allogeneic stem cell transplantation. T cells adapt to distinct stressors in order to meet their bioenergetic demand. After HSCT, the engagement of distinct metabolic pathways is correlated with T cell role and function. Glycolysis is the preferred pathway for GVHD-mediating alloactivated T naïve cells after exposure to host antigens, which differentiate them to their effector phenotype and increase the expression of Glut1 receptor. Glycolytic reactions also provide a bridge for macromolecule synthesis, redox balance and cell growth, by producing intermediate metabolites that favor PPP. The generation of pyruvate from glucose yields lactate in the cytoplasm or acetyl-CoA, which enters the TCA cycle in the mitochondria. In alloactivated GVHD-mediating T cells, expression of glucose, amino acid, and fatty acid transporters increase leading to enhanced entry of these compounds (thick arrows). The PI3K/Akt/mTOR pathway favors glycolysis and suppresses FAO. mTOR also potentiates PPP in these cells. GVHD-mediating T cells also use amino-acids, especially glutamine, which enters the cell by the increased expression of Slc7a5 transporters. Glutaminolysis yields α-ketoglutarate that enters the TCA cycle in the mitochondria although mitochondrial metabolism does not seem to be the dominant metabolic profile of alloreactive T cells. In T effector memory cells, the main T subset involved in mediating GVL function, FAO is the dominant energy source and mitochondrial metabolism is significantly enhanced, leading to OXPHOS reactions in the ETC, where higher amounts of ATP are produced, thereby providing sustained energy for cell survival. T regulatory cells are protective against GVHD, by inhibiting predominantly alloactivated GVHD-mediating T cells but inhibit GVL-mediating T memory cells to a lesser extent thereby preserving GVL. Treg cells share metabolic features with GVL cells, as they use FAO as their preferred energy source. mTOR regulation appears to have a distinct effect on Treg cells, as it is necessary for optimizing FAO that leads to adequate Treg differentiation and function. ADP, Adenosine diphosphate; ATP, Adenosine triphosphate; Akt, Protein Kinase B; ETC, Electron transport chain; FAD, Flavin adenine dinucleotide (oxidized state); FADH2, Flavin adenine dinucleotide (reduced state); G-6-P, Glucose-6-phosphate; G6PDH, Glucose-6-phosphate dehydrogenase; Glut1, Glucose transporter1; GVHD, graft-vs.-host diseases; GVL, graft-vs.-leukemia; HK-1/2, Hexokinase-1/2; HSCT, hematopoietic stem cell transplant; mTOR, Mechanistic/mammalian target of rapamycin; NAD, Nicotinamide adenine dinucleotide (oxidized state); NADH, Nicotinamide adenine dinucleotide (reduced state); NADP+, Nicotinamide adenine dinucleotide phosphate (oxidized state); NADPH, Nicotinamide adenine dinucleotide phosphate (reduced state); OXPHOS, Oxidative phosphorylation; PI3K, Phosphatidylinositol-4,5-bisphosphate 3-kinase; PPP, Pentose phosphate pathway; PK, Pyruvate kinase; Slc7a5, Solute carrier family 7 member 5; TCA cycle, Tricarboxylic acid cycle.

After HSCT, naïve donor T cells are directed to the recipient secondary lymphoid tissues, where they become activated by recipient's alloantigens (32, 33).When this happens, an increase in glycolysis and OXPHOS is induced (75–77). Overall, glycolysis escalates, becoming the principal source of energy for GVHD-causing T cells that under these conditions convert to T effector cells (75). For this mechanism to be efficient, carbohydrate catabolism mediators are also highly upregulated. These metabolic changes alter the functional profile of GVHD-mediating T cells, which are no longer naïve, but undergo differentiation to T effectors, simultaneously with metabolic reprogramming and proliferation in response to alloantigen-mediated stimulation (75) (Figure 2). In comparison to mice that received syngeneic BMT, mice undergoing allogeneic transplant displayed higher ECAR, accumulation of glycolytic intermediates, increased levels of LDH-A, Mct4, Glut1 and Glut3 mRNA, along with higher glucose-6-phosphate levels, all of which imply higher glycolytic activity (75). Consistent with the key role of glycolysis in regulating alloreactive T_EFF_ function, similarly to T_EFF_ of different specificity, overexpression of Glut1 results in superior T cell survival (78).

Glutamine metabolism is also a central component of T cell metabolic reprogramming during activation. T helper cell responses are supported by the upregulation of the glutamine/leucine transporter Slc7a5, and *Slc7a5* null cells are unable to complete metabolic reprogramming and fail to undergo differentiation and clonal expansion (79). The role of glutamine in T_EFF_ differentiation is supported by the observations that glutamine availability in the culture media increases IL-2 receptor expression, lymphocyte proliferation and cytokine production (79). Thus, glutamine is another critical source of energy and macromolecule production in activated T cells and might be involved in the development of alloreactive T cell responses and GVHD during alloHSCT (Figure 2).

Although mitochondrial metabolism has a role in the function of alloactivated T cells (76, 77, 80), it was also observed that regulators of fatty acid uptake and FAO are significantly reduced after autologous or allogeneic HSCT, compared to resting T cells. This correlated with metabolic reprogramming of alloreactive T cells to favor glycolytic metabolism and glutaminolysis as the key pathways for energy generation (75). FAO seems to increase in GVHD T_EFF_ cells only after the fifth cell division, around 3 days post BMT (76), suggesting that these metabolic pathways might have distinct roles during the life of alloreactive T_EFF_ cells *in vivo*. Nevertheless, most experimental evidence indicates that enhanced FAO is critical for T_MEM_ (27) and Treg cell activity (26, 72) and for this reason it would be protective against GVHD (12, 15, 16, 19). Thus, the precise role of FAO in alloreactive T cell function and the details of its regulation in GVHD remain to be determined.

Studies have indicated that administration of metformin, which activates AMPK thereby promoting FAO, might attenuate GVHD by supporting the differentiation of Treg and altering the balance between Th17 and Treg cells (81). This observation potentially provides an opportunity to repurpose metformin for the prevention or treatment of GVHD. However, two important issues should be taken into consideration: First, AMPK does not exclusively affect the function of Treg because T_EFF_ cells from AMPK KO mice display impaired differentiation and metabolic fitness, and impaired glutamine-dependent mitochondrial metabolism that allows T_EFF_ metabolic plasticity and survival under low-glucose conditions (61); Second, in addition to activating AMPK, metformin can inhibit complex I of the electron transport chain (ETC) (82) which may impact the metabolism and function of all T cell subsets independently of AMPK. Thus, glycolysis, FAO and AMPK remain attractive metabolic targets to explore for therapeutic immunomodulation of alloreactive T cells by individual or combinatorial approaches.

#### Microbiota in T Cell Metabolism and GVHD

The investigation of the role of microbiota in host immunity in health and disease is a highly active topic with major biological relevance. Commensal bacteria are closely related to the host's nutritional status and the function of the immune system. Our understanding about their role in disease pathogenesis is rapidly expanding. It is now well-known that the development, differentiation and polarization of T lymphocytes is affected by gut microbes (83, 84). Commensal microbe-derived SC-FA, butyrate and propionate, can promote the differentiation of Treg cells (85). In mice, *Clostridia* strains can induce CD4^+^Foxp3^+^ Treg differentiation by producing SC-FA (86). Tregs induced by these microbiota can also induce IL-10 and ICOS, affect the intestinal immune function, and prevent colitis and allergic diarrhea (86). Conversely, segmented filamentous bacteria in mice and *Bifidobacterium adolescentis* in humans, promote Th17 differentiation, enhance Th17 cell survival (87, 88) and exacerbate autoimmune arthritis (88). The latter reinforces the importance of understanding the harmful impact of symbiont-driven T helper cells in the context of inflammatory conditions.

Not unexpectedly, SC-FA can influence the development of GVHD (89). SC-FA regulate both T_EFF_ and Treg cells accumulation by increasing histone H3 acetylation in the locus of *Foxp3* and activating the mTOR pathway (90, 91). Consequently, Butyrate restoration in intestinal epithelial cells, implemented to overcome the reduction caused by the inflammatory cascade seen in alloHSCT, promotes histone acetylation and correlates with lower GVHD clinical scores (92). These findings indicate that microbial-derived metabolic products have a potential use in GVHD, probably due their impact on T cell subset differentiation and survival.

### Metabolism of Leukemia-Activated GVL Effector Cells

In mice, GVL effect is driven by CD4^+^ effector T_MEM_ that require cognate interaction with MHC-II and leukemia antigens (12). Although mouse models do not fully recapitulate human T_MEM_ life-long repertoire and CD4/CD8 ratio, it should be noted that the infusion of CD4^+^ T_MEM_ to recipients of T cell-depleted human allografts effectively enhanced GVL and immune reconstitution without increasing GVHD (93). This observation provided an important insight on the potential use of sorted T cell populations to promote GVL, instead of administering unfractionated donor lymphocyte infusions, which are associated with GVHD (94). The therapeutic efficacy of this approach was explored by implementing selective depletion of T naïve cells from allografts given to high-risk leukemia patients. This modification of the allografts resulted in comparable rates of aGVHD but significantly improved responsiveness of aGVHD to steroid treatment. In addition, these patients had decreased cGVHD rates and improved immune reconstitution characterized by rapid T cell recovery and transfer of protective anti-viral immunity (93). Thus, selective utilization of donor T_MEM_ cells might be the most preferred approach to preserve immunity while decreasing GVHD-mediated morbidity.

The reason for the differential action of T naïve and T_MEM_ cells after allogeneic HSCT, has been hypothesized to rely on their differential responses. Unlike T naïve cells, T effector memory alloreactive cells cannot expand or sustain high magnitude responses and while they are less likely to induce cGVHD, they are sufficient to mediate GVL function (12, 15). Moreover, cytokine production by memory T cells is also suboptimal, compared to naïve T cells that rapidly increase aGVHD-associated cytokines TNF-α, IL-1, IL-6, and IFN-γ or the cGVHD-associated cytokine IL-17 (95). Because T naive cells that convert to effectors and T memory cells engage different metabolic pathways to meet their energetic demands, the distinct nature of GVL-specific and GVHD-specific alloreactive T cell populations might provide an excellent opportunity to introduce selective metabolism-targeting therapies, to optimize GVL and prevent the development of GVHD. Moreover, the differentiation of Treg cells that have the ability to suppress GVHD but not GVL (16, 17) are also supported by metabolic pathways similar to those engaged by T_MEM_ cells such as oxidative metabolism and FAO (26, 54). This metabolic program of T_MEM_ cells is supported by utilization of LC-FA for FAO (54). Although the mitochondrial transporter of LC-FAs, Cpt1a, is involved in this mechanism (54), subsequent studies discovered that pharmacologic inhibition or genetic ablation of *Cpt1a* did not affect the generation of T_MEM_ (96), suggesting that T cells may metabolize short-chain fatty acid, in the absence of Cpt1a activity. Further, in *Cpt1a* KO T cells, the use the Cpt1a inhibitor, etomoxir, used in concentrations significantly higher than those required to inhibit Cpt1a, suppressed the generation of Tregs *in vitro*, suggesting an a Cpt1a-independent action (96). Thus, differentiation of T_MEM_ and Treg depends on FAO that is regulated by CPT1a-dependent and independent mechanisms. FAO might be a tentative therapeutic target to induce T_MEM_ and Treg differentiation in order to prevent GVHD and preserve GVL.

### Metabolism of Antigen Presenting Cells and Relevance to Allogeneic-HSCT

The role of APCs, both from host and donor, in the setting of GVHD and GVL is a growing research focus during the past few years, given the recent understanding of their key role in both processes in alloHSCT (33, 97, 98). Recipient APCs are also important mediators of graft rejection, due to their potential to activate graft-infiltrating T cells (99). Today, it is well-known that the activation of the innate arm of the immune system is essential for the unfolding of GVHD, as APCs mediate T-cell priming and imprinting to GVHD target organs after transplantation (30, 98). Professional APCs, comprised DCs, B cells and macrophages, are capable of processing and presenting antigens to T cells through MHC proteins, promoting the formation of the immunological synapse that allows development of adaptive immune responses (98). During HSCT, host bone marrow APCs are mostly ablated by the conditioning regimen (100). Under these circumstances, skin macrophages and, to a lesser extent, dendritic cells engage in most of the antigen-presenting activities, mainly due to their resistance to myeloablative regimens (101, 102). Host and donor APCs have different roles in the development of GVHD. Host APCs seem to be involved in the induction of aGVHD, while donor macrophages contribute to cGVHD by cross-priming alloactivated CD8^+^ T cells (98, 103, 104). Replacing host APCs with donor APCs reverses T cell activation, as it decreases the interaction between GVHD-related host APCs and donor CD8^+^ T cells (28). Additionally, depletion of host liver and spleen-resident APCs results in decreased recruitment of allogeneic CD8^+^ T cells, thereby suppressing hepatic aGVHD but not skin involvement (29). Paradoxically, host APCs also take part in GVL function, whereas donor APCs only have a limited role in this process (105).

APCs activate different metabolic pathways, depending on the engagement of specific accessory surface receptors, cytokine stimulation and other microenvironmental cues. DCs increase their glycolytic activity upon their TLR activation as a means to produce enough pyruvate that can activate TCA cycle reactions and OXPHOS (3, 106). IFN-γ-mediated signals can direct macrophages into the classic M1 proinflammatory phenotype, in which glucose uptake via GLUT1 mediated influx predominates. LPS expressed on the outer membrane of gram-negative bacteria, after interacting with TLR4 on M1 macrophages, induces glycolysis, leading to lactate accumulation and production of TCA cycle metabolites, particularly succinate, which induces the IL1-β production and inflammation (107). Conversely, IL-10, IL-4, and IL-13, induce the alternative anti-inflammatory M2 phenotype, which relies mostly on mitochondrial respiration and instead of inducing tissue inflammation, promotes resolution of inflammation, tissue remodeling, and repair (108–110).

These extensive studies indicate that the metabolic profile of M1 macrophages has similarities to that exhibited by activated effector-like T cells (such as those inducing GVHD), while the metabolic phenotype of M2 macrophages parallels that of memory-like T cells (such as those inducing GVL). Thus, concomitant metabolic reprogramming of APCs and T cells will have an important role in the net outcome of GVHD and GVL and these outcomes might vary dependent on the metabolic polarization of one or both these immune populations. For instance, the preferential engagement of APCs and T cells in glycolytic metabolism will allow immune cells to sustain inflammatory GVHD-mediating functions by promoting the generation of GVHD-inducing M1 macrophages and effector-like T cells. Conversely, the metabolic shift of these cell populations toward oxidative phosphorylation might selectively promote the differentiation of GVL-inducing memory-like T cells while supporting M2 differentiation and resolution of inflammation thereby preventing or suppressing GVHD. Indeed, inhibition of FAO by etomoxir suppressed M2 polarization of macrophages (111) and T_MEM_ cell differentiation in a Cpt1a dependent and independent manner (96). It should be noted that immune cell polarization *in vivo* is not an all or nothing event but rather a continuum that leads to an immune signature depending on the dominating metabolic balance, thereby providing an opportunity for therapeutic intervention through implementation of subtle metabolic changes that will influence both APCs and T cells. Future studies are needed to investigate whether targeting glycolytic metabolism will have a similar simultaneous effect to suppress both M1 macrophage polarization and generation of alloreactive T effector cells that mediate GVHD.

## Therapeutic Relevance of Metabolism in Allogeneic Hematopoietic Stem Cell Transplantation

The central goal of post-transplant therapeutic immunomodulation is the prevention or treatment of GVHD, without diminishing GVL activity. As outlined in the previous sections, the distinct metabolic pathways in these processes point to potential new therapeutic targets. Although FAO and glutamine metabolism might have a role in the activation of GVHD-inducing alloreactive T cells, elegant work has provided evidence that glycolysis is the dominant metabolic pathway of alloactivated donor T cells and inhibition of glycolysis by targeting HK-2 or the rate-limiting PFKFB3 prevents alloreactivity *in vivo* and attenuates GVHD in HSCT recipient mice (75). Blocking PFKFB3 in cancer cells also downregulates glucose influx, thereby interfering with tumor growth and disease progression (112). Thus, targeting PFKB3 might control GVHD while suppressing metabolic activity and growth of residual leukemia cells. Other means of reducing glycolytic activity also translate into amelioration of GVHD severity. The role of glucose metabolism in GVHD is also supported by the observation that Glut1 transporter-deficient murine CD4^+^ effector T cells are unable to expand and induce GVHD *in vivo*, while, in this context, Treg population increases, showing independence from Glut1 (113). Notably, inhibiting glycolysis by using 2-deoxyglucose, not only diminished the expansion of T_EFF_ cells but enhanced the differentiation of CD8^+^ T_MEM_ cells (114). Similarly, IL-15-driven overexpression of Cpt1a induced T_MEM_ cell production and supported their survival (54). Such modifications in the abundance of T cell subsets by targeting glycolysis might selectively prevent GVHD while preserving GVL.

Targeting PI3K/AKT/mTOR pathway has been explored in the context of alloHSCT, because this pathway is central to the activation, expansion and differentiation of T_EFF_, T_MEM_, and Treg cells. Inhibition of PI3K/AKT/mTOR with BEZ235, a dual PI3K/mTOR inhibitor, resulted in decreased T cell activation and diminished GVHD grade (115). Importantly, using rapamycin, an mTORC1 inhibitor, enforced FAO and increased T_MEM_ cell differentiation (116). Because rapamycin also promotes the differentiation of Treg (69), such approach might selectively suppress GVHD and promote GVL by inducing T_MEM_ and Treg. Thus, rapamycin might be repurposed and used not simply as an immunosuppressant but also as an immunomodulator to alter metabolism-driven differentiation of T cell subsets in recipients of alloHSCT.

Utilization of mitochondrial F1F0-ATPase inhibitor, Bz-423, promotes apoptosis of alloactivated cells, thereby reducing GVHD rates and improving survival without impairing immune reconstitution (77). The Cpt1a inhibitor, etomoxir, was also reported to decrease GVHD severity in mice after day 30 post-transplant, without impairing immune reconstitution (76). As mentioned above, etomoxir, used in concentrations significantly higher than those required to inhibit Cpt1a, suppressed the generation of Tregs *in vitro*, suggesting a Cpt1a-independent action (96). Such Cpt1a-independent effect of etomoxir was also observed in bone marrow derived macrophages from Cpt1/2 KO mice, in which etomoxir retained the ability to disrupt IL-4-mediated M2 macrophage polarization possibly by causing depletion of intracellular coA (111). Thus, the combined effects of such metabolism-targeting compounds might have implications in the components of the innate and adoptive immune system resulting in clinical effects on GVHD, GVL and immune reconstitution that are driven by the altered function of more than one immune population or by previously unidentified selective effects on a certain immune cell population. This is also supported by the observation that AEB071, an inhibitor of protein kinase C-θ that preferentially halts Treg differentiation and activation, preserves graft survival and GVL but prevents IFN-γ production and GVHD by enhancing the function of Treg (117).

Together, the results of targeting studies in various mouse models (54, 75, 77, 113, 115–117) are of direct clinical relevance and indicate that therapeutic targeting of selective components of signaling and metabolic pathways might have distinct outcomes on T cell differentiation and distinct clinical implications in the prevention and treatment of GVHD and GVL. Because possibly distinct metabolic mechanisms dominate during different phases of alloreactive T cell lifespan, it will be critical to determine the metabolic signatures of alloreactive GVHD- and GVL-specific T cells during various times after alloHSCT. Such knowledge will allow the design of proper therapeutic combinatorial therapies to selectively induce the desired metabolism-driven immune cell differentiation.

A major challenge when targeting metabolism for therapy of GVHD, will be to preserve the metabolic properties of pathogen-specific T_EFF_ cells, which are mandatory for their function under conditions of stress and response to pathogens. Future work is required to identify and dissect the potential metabolic differences of pathogen-specific vs. host antigen-specific T cells that induce GVHD. Identifying pathways that dominate in each of these populations during their lifespan will allow the development of experimental approaches and clinical trials to implement and evaluate metabolic interventions in these distinct T_EFF_ cell populations in parallel to studies dissecting the effects of such approaches on GVHD vs. GVL.

## Concluding Remarks

Metabolism is a rapidly growing subject in immunology and malignant hematology. LSC that survive under intensive chemotherapy are responsible for MRD and relapse. These LSC use both anabolic and catabolic pathways, depending on the environmental cues. Our knowledge regarding the metabolic features of leukemia cells in relapsed or resistant disease in patients who undergo allogeneic HSCT is limited because relevant studies are currently missing. Based on current data, relapsed or resistant leukemia cells display features of LSC, which are highly dependent on glycolysis, but also have the metabolic plasticity to adopt other metabolic programs for energy generation, including mitochondrial metabolism, FAO and autophagy. Thus, although therapeutic approaches to target glycolytic metabolism employed to suppress GVHD-mediating T_EFF_ cells are expected to suppress or eradicate leukemia cells, it is possible that the high degree of plasticity and metabolic adaptation of LSC may provide them the means to survive by shifting their metabolic preferences. Identifying LSC dominant pathways upfront and modulating them by metabolism-targeting interventions, together with chemotherapy, will be highly beneficial because will eradicate LSC, thereby minimizing the risk for relapse. It is particularly intriguing and hopeful to attempt achieving this objective, together with selective metabolism-driven differentiation of immune cell subsets, with the goal to minimize GVHD and enhance GVL after allogeneic HSCT.

## Author Contributions

NT-O: wrote the main body of the manuscript and prepared figures; TK: wrote several sections of the manuscript and provided relevant citations; H-TW: wrote sections of the manuscript and provided relevant citations; VB: supervised NT-O, TK, and H-TW and was responsible for the overall preparation of the manuscript. All authors read and approved the content of the manuscript.

### Conflict of Interest Statement

The authors declare that the research was conducted in the absence of any commercial or financial relationships that could be construed as a potential conflict of interest.

## Abbreviations

ACC1: acetyl-CoA carboxylase 1
acetyl-CoA: acetyl coenzyme A
AEB071: Sotrastaurin
aGVHD: Acute graft-vs.-host-disease
ALL: Acute lymphoblastic leukemia
AML: Acute myelogenous leukemia
alloHSCT: Allogeneic hematopoietic stem-cell transplantation
AKT: Protein Kinase B
AMPK: AMP-activated protein kinase
APC: Antigen-presenting cell
ATP: Adenosine triphosphate
BEZ235: Dactolisib
BCL-2: B-cell lymphoma 2
Bz-423: Benzodiazepine (Bz)-423
C75: *trans*-4-Carboxy-5-octyl-3-methylene-butyrolactone
cGVHD: Chronic graft-vs.-host-disease
CML: Chronic myelogenous leukemia
CoA: Coenzyme A
CPT1a: carnitine palmitoyl transferase
DC: Dendritic cell
ECAR: extracellular acidification rate
F1F0-ATPase: F1 portion and a transmembrane FO portion of ATP synthase
FADH_2_: Flavin adenine dinucleotide
FAO: Fatty acid oxidation
FAS: Fatty acid synthesis
FOXP3: Forkhead-Box P3
Glut1: Glucose transporter 1
Glut 3: Glucose transporter 3
GVL: Graft-vs.-leukemia
GVHD: Graft-vs.-host-disease
HIF-1α: Hypoxia-inducible factor 1α
HK2: Hexokinase 2
HSC: hematopoietic stem cells
HSCT: hematopoietic stem cell transplantation
ICOS: Inducible T-cell costimulator
IFNγ: Interferon γ
IL-1/2/4/6/7/10/13/15: Interleukin 1/2/4/6/7/10/13/15
iTreg: inducible regulatory T cell
KO: knock out
LC-FA: Long-chain fatty acids
LPS: Lipopolisacharide
LSC: leukemia stem cells
LDH-A: Lactate dehydrogenase-A
M1: Classically activated macrophage
M2: Alternatively activated macrophage
MHC: Major Histocompatibility Complex
MHC-II: Major Histocompatibility Complex type 2
MM: Multiple myeloma
MRD: Minimal residual disease
MP: Myeloid progenitors
Mct4: Monocarboxylate transporter 4
mTOR: Mechanistic/mammalian target of rapamycin
mTORC1: Mechanistic/mammalian target of rapamycin complex 1
mTORC2: Mechanistic/mammalian target of rapamycin complex 2
Myc: Myc proto-oncogene
NADH: Nicotinamide adenine dinucleotide
NADPH: Nicotinamide adenine dinucleotide phosphate
NFAT: Nuclear factor of activated T cells
NOS: Nitric oxide synthase
OXPHOS: Oxidative phosphorylation
PFKFB3, 6-phosphofructo-2-kinase/fructose-2: 6-biphosphatase 3
PI3K, Phosphatidylinositol-4: 5-bisphosphate 3-kinase
PKM2: Pyruvate kinase M2
PPP: Pentose phosphate pathway
ROS: Reactive oxygen species
SREBPs: Sterol regulatory element-binding proteins
SC-FA: Short-chain fatty acids
SLC7A5: Solute Carrier Family 7 member 5
TCA cycle: Tricarboxylic acid cycle
TCR: T cell receptor
T_EFF_: T effector cells
Th: T helper cells
Th1: T helper 1
Th2: T helper 2
Th17: T helper 17
Tfh: follicular T helper
TLR: Toll-Like Receptor
TKI: Tyrosine kinase inhibitors
TME: Tumor microenvironment
T_MEM_: T memory
TNF α: Tumor necrosis factor α
Treg: Regulatory T cells.
